# Double-sided slippery liquid-infused porous materials using conformable mesh

**DOI:** 10.1038/s41598-019-49887-3

**Published:** 2019-09-16

**Authors:** Nicasio R. Geraldi, Jian H. Guan, Linzi E. Dodd, Pietro Maiello, Ben B. Xu, David Wood, Michael I. Newton, Gary G. Wells, Glen McHale

**Affiliations:** 1 School of Science and Technology, Nottingham Trent University, Nottingham, NG11 8NS UK; 2 Smart Materials and Surfaces Laboratory, Faculty of Engineering and Environment, Northumbria University, Newcastle upon Tyne, NE1 8ST UK

**Keywords:** Nanoparticles, Fluids, Surfaces, interfaces and thin films

## Abstract

Often wetting is considered from the perspective of a single surface of a rigid substrate and its topographical properties such as roughness or texture. However, many substrates, such as membranes and meshes, have two useful surfaces. Such flexible substrates also offer the potential to be formed into structures with either a double-sided surface (e.g. by joining the ends of a mesh as a tape) or a single-sided surface (e.g. by ends with a half-twist). When a substrate possesses holes, it is also possible to consider how the spaces in the substrate may be connected or disconnected. This combination of flexibility, holes and connectedness can therefore be used to introduce topological concepts, which are distinct from simple topography. Here, we present a method to create a Slippery Liquid-Infused Porous Surface (SLIPS) coating on flexible conformable doubled-sided meshes and for coating complex geometries. By considering the flexibility and connectedness of a mesh with the surface properties of SLIPS, we show it is possible to create double-sided SLIPS materials with high droplet mobility and droplet control on both faces. We also exemplify the importance of flexibility using a mesh-based SLIPS pipe capable of withstanding laminar and turbulent flows for 180 and 90 minutes, respectively. Finally, we discuss how ideas of topology introduced by the SLIPS mesh might be extended to create completely new types of SLIPS systems, such as Mobius strips and auxetic metamaterials.

## Introduction

Recent advances in liquid shedding materials have seen the development of Lubricant Impregnated Surfaces (LIS)^1^ or equivalently Slippery Liquid-Infused Porous Surfaces (SLIPS)^2^ inspired by the *Nepenthes* pitcher plant. These are, by definition, porous or textured surfaces into which a layer of lubricating liquid is imbibed and stabilized by capillary forces. Conceptually, these surfaces have many similarities to their conventional superhydrophobic counterparts. They both rely on a lubricating layer, whether that be air or a liquid, to reduce or remove the contact between the droplets and the underlying solid surface. The fundamental distinction of these surfaces is the possibility of using a highly wetting liquid lubricant, which remains completely wetting when immersed in, or contacted by, another immiscible liquid (typically water). On a superhydrophobic surface a lubricating air layer would be displaced, which is not the case with SLIPS^3^. The liquid lubricant layer thus eliminates the contact line pinning normally associated with sessile droplets on solid surfaces, leading to high drop mobility and remarkably low contact angle hysteresis^4^. In addition to superhydrophobicity and SLIPS, the only other materials method, of which we are aware, to eliminate contact line pinning is to use a Slippery, Omniphobic, Covalently Attached Liquid (SOCAL) surface^5,6^.

The first demonstrated SLIPS came from Wong *et al*. in 2011. The surfaces comprised of electrospun Teflon mats, which were chemically modified and impregnated with fluorinated oil^2^. They also demonstrated SLIPS using a perfluorinated oil infused Teflon membrane, although they did not report on its flexibility or discuss the possibility of utilizing the two surfaces of the membrane. Others have also shown it is possible to create SLIPS using nanofiber mats^7–9^, lithographically made structures^1,4,10–13^, electrodeposition^14,15^, porous films^16,17^, roughened surfaces^18–20^, nanoparticles^21–23^, and colloid templating^24^. The focus has tended to be on creating the surface area with suitable wetting properties to stabilize the infusing liquid in the presence of the droplet and to obtain low droplet sliding angles. Moreover, these methods are often limited to simple geometries and flat planar surfaces. Current methods to create flexible SLIPS surfaces are not able to achieve water sliding angles less than 5.5°^17^. Furthermore, they are limited to single-sided substrates.

Double-sided, flexible and conformable meshes would allow SLIPS technologies to access complex surface geometries whilst sustaining flow and water repellency. There are many materials, such as membranes and foils, which inherently have two surfaces of relevance. Interestingly, these surfaces can be topologically complex, such as with meshes that have disconnected holes laterally whilst providing connection for an infusing oil between the two surfaces. The concept of topology is quite different to that of topography, which is the dominant concept when using lithographic or etching techniques to create the texture needed for SLIP surfaces. The porosity of membranes can be thought of as an alternative method to create the increased surface area needed for an infusing oil to coat and be retained by a surface due to the surface energetics. However, when there are two surfaces and materials with holes and spaces, the concept of topology can become important and this is a global rather than local geometrical concept. For example, a doughnut and a sphere both have one surface, which appears on a local scale to be the same, but globally the doughnut has a hole which a sphere does not. The topological properties, such as holes and connectedness, are relevant to a SLIP surface because for it to self-heal an infusing liquid must flow between different spaces in the structure. In this work, we focus on a flexible double-sided mesh and these concepts are important as they relate to how the two surfaces are connected.

In our meshes, the flexibility of the material can be used to create macroscopic shapes by rolling them into tubes for flow or folding them to conform to irregular shaped containers. Furthermore, a double-sided mesh can easily be twisted and folded to create highly complex macro structures. This gives the potential for combing the idea of SLIPS with the ideas of auxetic^25^, and Origami and Kirigama-based materials^26–29^. In the auxetic case, a material can have the counter-intuitive property of becoming wider when stretched (i.e. it possesses a negative Poisson’s ratio,ν) enabled by the structure of its lattice which means all expansion occurs *via* its holes. Another intriguing possibility is a double-sided membrane in the form of a tape into which a single twist of 180° of one end could be introduced prior to joining its ends to create a SLIPS Mobius strip^30^. The ability to create complex macro structures from double-sided SLIP meshes also allows for the study of fluids in larger scale scenarios such as internal and external fluid flows through or around objects and to investigate the potential applications of flexible porous materials with tailored surface properties^31–33^.

In this paper, we illustrate one potential application of highly flexible and durable mesh SLIPS, by demonstrating SLIP mesh pipes, which are capable of withstanding turbulent flows for a significant duration of time. We introduce a method to create a SLIP surface for this purpose using a combination of surface texturing and chemical functionalization by spray coating a fractal-like porous network on the solid surface, which, when impregnated with silicone oil, forms mesh SLIPS. We first show that this method can minimize contact angle hysteresis on both flat and curved surfaces. We then show that the same method can be used to create double-sided mesh SLIPS which can be bent, twisted and folded, without loss of their lubricating properties. Moreover, this original method for the creation of double-sided SLIPS materials can be generally applied to any substrate, including meshes, foils and membranes, and then allows it to be infused with a lubricating oil, without the need for the substrate material to be hydrophobic. As a mesh is composed of interwoven wires, the holes between the wires allows the lubricating fluid to transfer between the faces of the material allowing the SLIPS materials to self-repair both faces. A SLIPS-based mesh is also an exemplification of a flexible substrate which can be formed into an unusual surface, such as a Mobius strip. Finally, we discuss the potential for combining SLIPS with the principles of auxetic, Origami and Kirigami-based meta-materials. These concepts have never previously been introduced in the literature in the context of SLIP surfaces.

## Results and Discussion

An ideal SLIPS surface must satisfy three criteria. First, the lubricating liquid must fully imbibe into and wet the surface structure. Secondly, the chosen working fluid must not displace the lubricating liquid when introduced to the surface and, thirdly, the two liquids must be immiscible^1^.

One way to produce a surface into which the lubricating fluid will fully imbibe is to produce an oleophilic rough coating. Many superhydrophobic coatings are also oleophilic due to the interaction of the rough surface with high and low surface tension liquids. Water has a relatively high surface tension, whereas oils tend to have a low surface tension. This often means that when a surface is rendered superhydrophobic, the tailored roughness enhances the oleophilicity of the surface^34,35^. The presented coating comprises of a superhydrophobic/superoleophilic micro-scale porous network of functionalized nanoparticles, which fulfils the first criteria for creating a SLIPS when impregnated with silicone oil. This is confirmed by the complete spreading and wetting of the surface by the lubricating silicone oil when applied to the coated surface. The use of silicone oil also satisfies the second condition, as silicone oil and water are immiscible.

**Figure 1:**
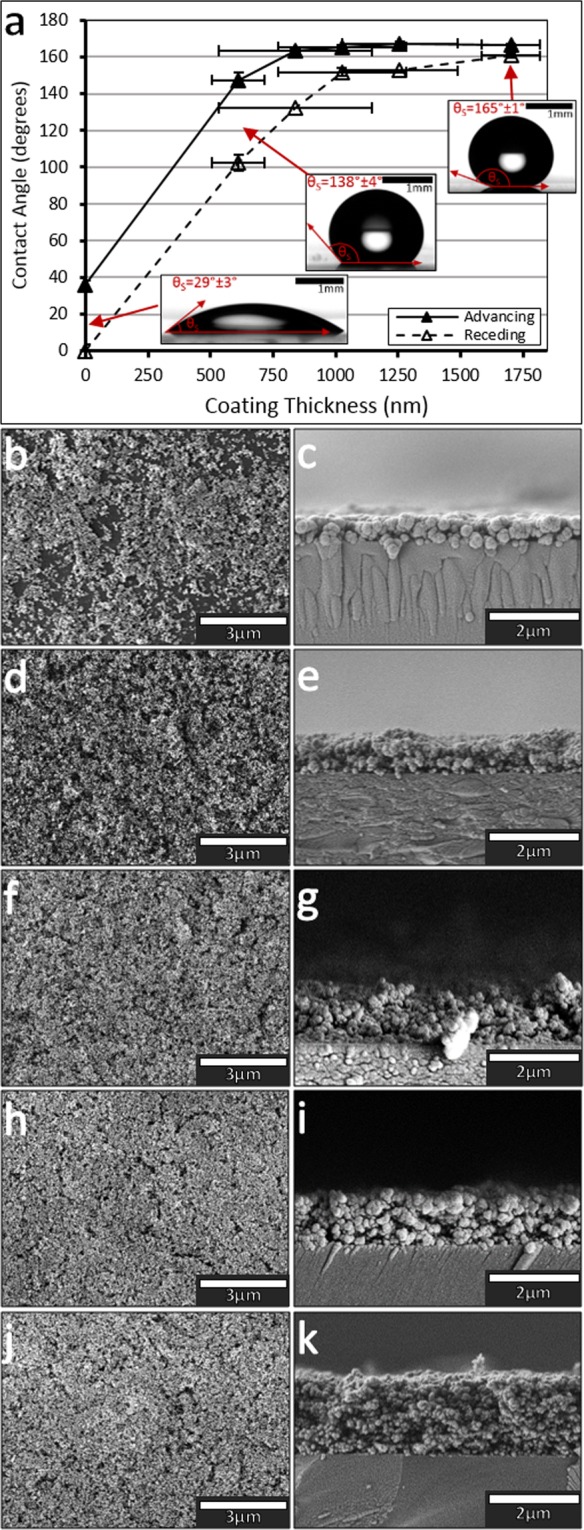
(**a**) The advancing and receding contact angles for substrates with increasing number of coatings. SEM top down images were also taken of 1 to 5 spray coatings, (**b**,**d**,**f**,**h**,**j**) respectively. SEM cross-section images were also taken for 1 to 5 spray coatings, (**c**,**e**,**g**,**I**,**k**), respectively.

**Figure 2:**
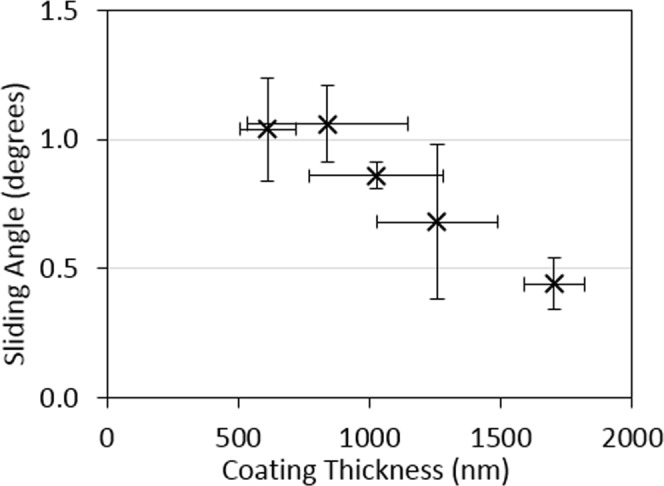
Sliding angles (X) for 2 µl deionized water droplets as a function of nanoparticle coating thickness on glass.

The first stage when creating the lubricant impregnated surface is to create the rough hydrophobic surface coating. Figure 1(a) shows the advancing and receding contact angles for glass substrates as a function of the coating thickness. After one application of the nanoparticulate coating, resulting in a coating thickness of 612 ± 112 nm (Figs 2, 1(b,c)), the surface becomes hydrophobic, as exemplified by the increase in advancing and receding contact angles, from 36.0 ± 2.5° to 147.5 ± 3.9° and from ~0° to 102.4 ± 7.4°, respectively. Despite the increase in contact angle, the surface coverage is heterogeneous, resulting in localised pinning sites, as seen in Fig. 1(b).

By applying up to five layers of the nanoparticulate coating, complete coverage of the substrate, with a continuous microscopic porous network, is achieved. This layer forms a superhydrophobic surface with low hysteresis of ~6°, displaying advancing and receding contact angles of 166.7 ± 0.2° and 160.7 ± 1.0°, respectively. The average thickness of the layer is increased to 1703 ± 116 nm (Fig. 1(d–k)).

**Figure 3:**
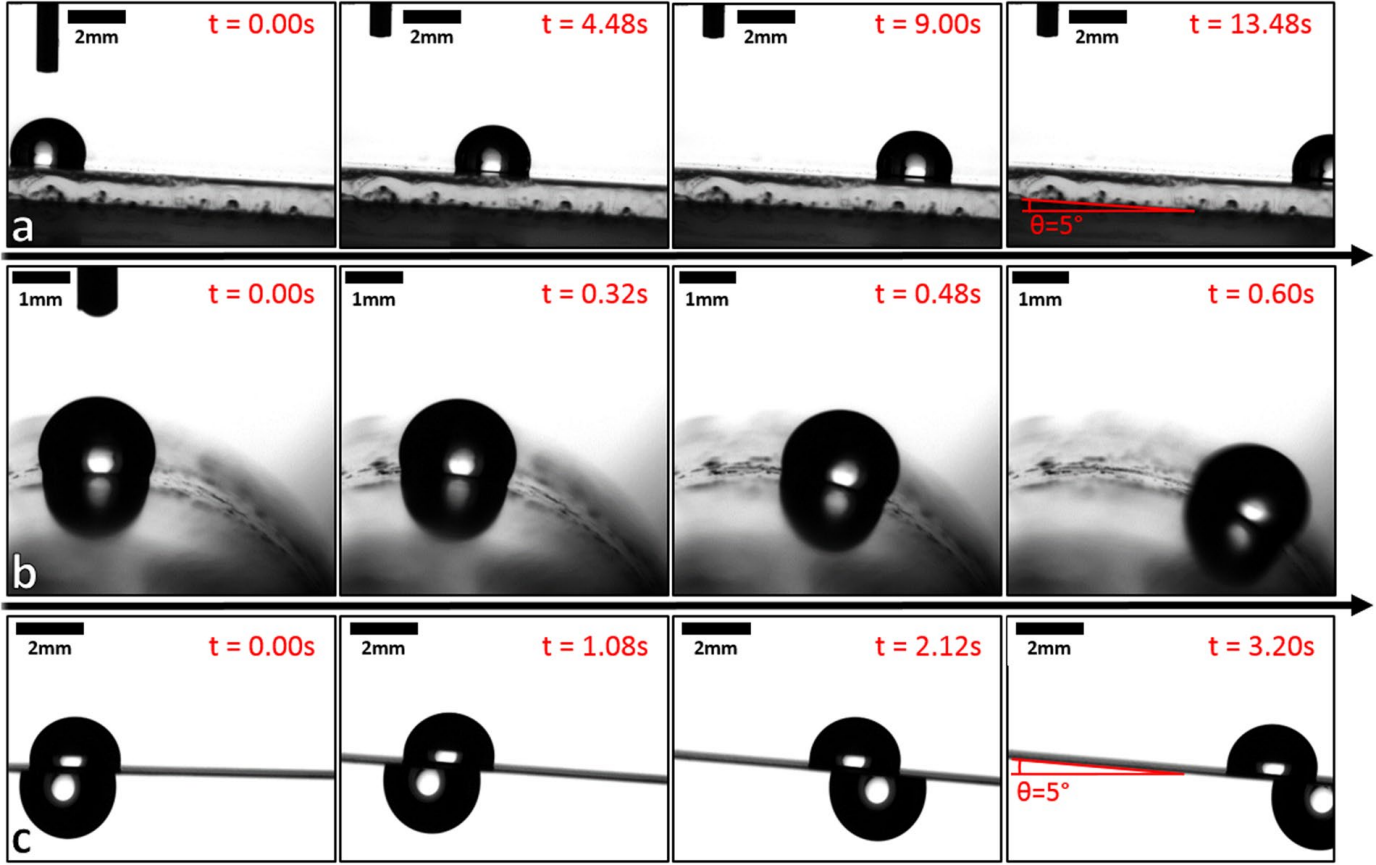
Time sequence of ~2 µl distilled water droplets on (**a**) a flat glass SLIP surface with a tilt angle of 5° from horizontal, (**b**) a glass tube SLIP surface, and (**c**) on both sides of a stainless steel mesh SLIPS, whilst the tilting angle of the mesh was increased to 5° from horizontal.

After dip coating in silicone oil the sliding angles were measured, with the results displayed in Fig. 2. The sliding angles for all samples are below 1.5°. This satisfies the requirement for SLIP surfaces to have sliding angles less than 5°. Figure 3(a) illustrates how a droplet of water slides on the glass SLIP surface, inclined at 5° from horizontal. With increasing number of coatings from one to five, the sliding angle of the glass SLIP surfaces falls from 1.04 ± 0.20° to 0.44 ± 0.10°.

Due to the simplicity and versatility of this method to render surfaces SLIPS, it can be extended to treat other systems that rely on a low hysteresis surface, e.g. for systems that rely on capillary forces in the investigation and manipulation of droplet motion^4,36,37^.

The application of the nanoparticle coating provides both a superhydrophobic and superoleophilic substrate. The superhydrophobicity repels water from the surface, whilst the porous superoleophilic surface layer stabilizes the lubricating silicone oil. The combination of the hydrophobic/oleophilic porous coating and the stabilized lubricating silicone oil means that the three criteria for producing a SLIPS are fulfilled, as the silicone oil fully imbibes into and wets the surface, water does not displace the stabilized silicone oil, and the silicone oil and water are immiscible.

To demonstrate the simplicity and versatility of this SLIPS production method, SLIPS were fabricated on increasingly complex structures. Figure 3(b) shows a time sequence of a ~2 µl distilled water droplet on a SLIPS coated 10 mm diameter glass tube, displaying very low sliding angles.

By applying the SLIPS coating to stainless steel meshes, it is possible to create slippery liquid infused materials, where both sides of the flexible material display low frictions properties (Fig. 3(c) and Supplementary Videos 1 and 2). The ability to coat flexible conformable meshes demonstrates the ability of the coating method to coat structures of increased complexity.

**Figure 4:**
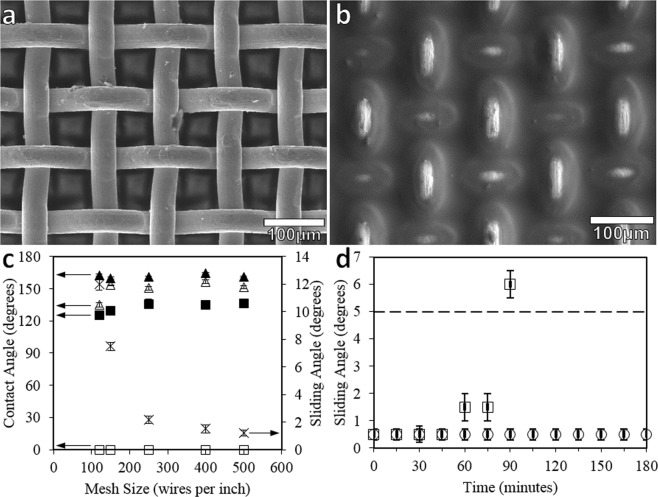
SEM images of an (**a**) #250 uncoated stainless steel mesh and (**b**) #250 stainless steel mesh SLIPS. (**c**) Advancing (filled) and receding (unfilled) contact angles for uncoated (■,□) and nanoparticle-coated (▲,Δ) stainless steel meshes, including sliding angles (X) for mesh SLIPS. (**d**) Sliding angles for 2 µl water droplets over time for #250 mesh SLIPS in laminar (~1500 Re)(○) and turbulent (~4000 Re) (□) water flows.

A range of stainless steel meshes were coated and infused with silicone oil, from #120 to #500, to determine the droplet sliding properties for different sized meshes (Fig. 4). This mesh structure has square open voids between the wires, meaning the material is porous, so fluids and gas can easily pass through the unmodified material. When the SLIPS coating is applied to the meshes, the silicone oil both coats the wires and fills the voids between the wires. This was confirmed using low-vacuum SEM (Fig. 4(a,b)). With both sides of the material rendered SLIPS, the voids in the material allow the silicone oil to flow between the two sides, thus providing a double-sided self-healing mechanism.

The contact angles for the nanoparticle-coated meshes are shown in Fig. 4(c). All the meshes displayed uncoated advancing contact angles of between 125° to 137° and uncoated receding contact angles of 0°. After the application of the nanoparticle coating the advancing and receding angles are increased. All advancing angles for the nanoparticle coated meshes are above 159°, with all receding angles above 150°, apart from the #120 mesh which only has a receding angle of 135° ± 2°. The large wire diameter of 90 μm with a wire separation of 122 μm of the #120 mesh is likely to be the origin of its smaller receding angle. We therefore conclude, the nanoparticle-coating transforms the #150 to #500 meshes into superhydrophobic materials.

After infusion with lubricating oil, the sliding angle for the 5 sized meshes was determined (Fig. 4(c)). The sliding angle for the #250, #400 and #500 mesh SLIPS are less than 5°, for 2 µl water droplets, so fall within the requirements for SLIPS surfaces. The lowest sliding angle was 1.2 ± 0.2° and was recorded for the #500 mesh SLIPS, which was the smallest available to us which could also be obtained at sufficient scale for the flow experiments.

The robustness of the slippery liquid infused materials was tested in a flow situation, where the #250 mesh SLIPS was subjected to a constant flow of water at Re = 8200 ± 500. Different samples were fabricated with 20 cSt and 1000 cSt silicone oil as the lubricating liquid. It was determined the 20 cSt mesh SLIPS could maintain a sliding angle of <5° for a maximum of 33 minutes before sufficient degradation was caused to the surface coating, rendering the material non-SLIPS. The 1000 cSt mesh SLIPS samples were comparably more robust, being able to maintain sliding angles less than 5° for up to 90 minutes.

After subjecting the 1000 cSt mesh SLIPS to a laminar flow of Re = 1550 ± 50, the sliding angle of water droplets on the surface did not deviate from 0.5 ± 0.2°, for 10 µl water droplets, over the course of 180 minutes, as shown in Fig. 1(d). However, in a turbulent flow of Re = 4000 ± 300, the sliding angle of water droplets remained low at 0.5 ± 0.2° for 45 mins before rising to 6.0 ± 0.5° at 90 minutes. This indicates that, when combined with the #250 mesh the SLIPS coating is able to withstand the shear stress generated by the laminar flow of water over the surface to a far greater extent than those generated by the turbulent flow of water.

Finally, we return to the general concept of topology and the potential for creating novel surfaces and materials. Figure 3(c) with droplets in view on both the upper and lower surfaces illustrates the ability of a SLIPS mesh to provide a double-sided SLIP surface. Such a double-sided material in a tape form could have a single 180° twist inserted, using the flexibility of the material, and the ends joined to create a single-sided SLIPS mobius strip^30^. In such a case, a drop translated once around a circuit of the strip would move from the equivalent of the “top” in such a figure to the “bottom” but would in fact remain on the same single side of the Mobius strip. To further illustrate new materials possibilities, one can consider the design of the holes in a mesh. Axially stretching a connected set of holes each defined by a six-sided hexagonal honeycomb network causes the transverse dimension to shrink. Often there may be an implicit assumption that stretching a material causes it to thin. However, if the connected holes are in the shape of, e.g., a re-entrant honeycomb network based on bow-tie elements, an auxetic material^25,38^, with a negative Possion’s ratio, which increases its thickness when it is stretched, can be created. Since it is possible to create auxetic materials using three-dimensional structures these ideas are not limited to surfaces arising from meshes and membranes. Thus, extending the concept of flexibility, which has potential for SLIPS-based Origami materials, to stretchability of meshes or other materials with specifically designed holes, one can now imagine creating SLIPS-based auxetic metamaterials.

## Conclusion

In this paper, we have presented mesh SLIPS as an exemplar of the concepts of flexible slippery double-sided membranes and to explore topological concepts for SLIPS. These meshes were fabricated through the application of a simple SLIPS coating method, onto the complex geometry of a mesh, which does not involve complex fabrication techniques and costly materials. This method uses a hydrophobic nanoparticle spray coating that can be applied to various surfaces and forms a hydrophobic/oleophilic porous structure, which can then be impregnated with a lubricating oil. On smooth surfaces this method can achieve sliding angles as low as 0.44 ± 0.10°. By applying the coating method to highly complex metallic meshes, which are flexible, conformable, and double-sided, mesh SLIPS were created, with low sliding angles of 1.2 ± 0.2°. When applied to a #250 stainless steel mesh, with a 1000 cSt silicone oil, the material was able to maintain a low sliding angle below 2° for up to 75 mins in turbulent flows and up to 180 mins in laminar flows.

This work validates that a double-sided conformable SLIP surface has been achieved with evidenced robustness as shown through mesh-based pipes withstanding both laminar and turbulent flows for a significant amount of time. The ability to create flexible slippery double-sided surfaces, such as meshes, to create slippery coatings on complex surfaces, and to conceptualize topological ideas will open-up the field of research in the use of slippery surfaces. To illustrate future possibilities, we have discussed how SLIPS Mobius strips, SLIPS auxetic, and Origami and Kirigami approaches may be possible to create new types of materials with slippery surfaces. These methods and ideas will help research to move beyond the standard characterization of the properties of slippery surfaces and allow much greater access into the applications of slippery materials.

## Experimental Methods

**Figure 5:**
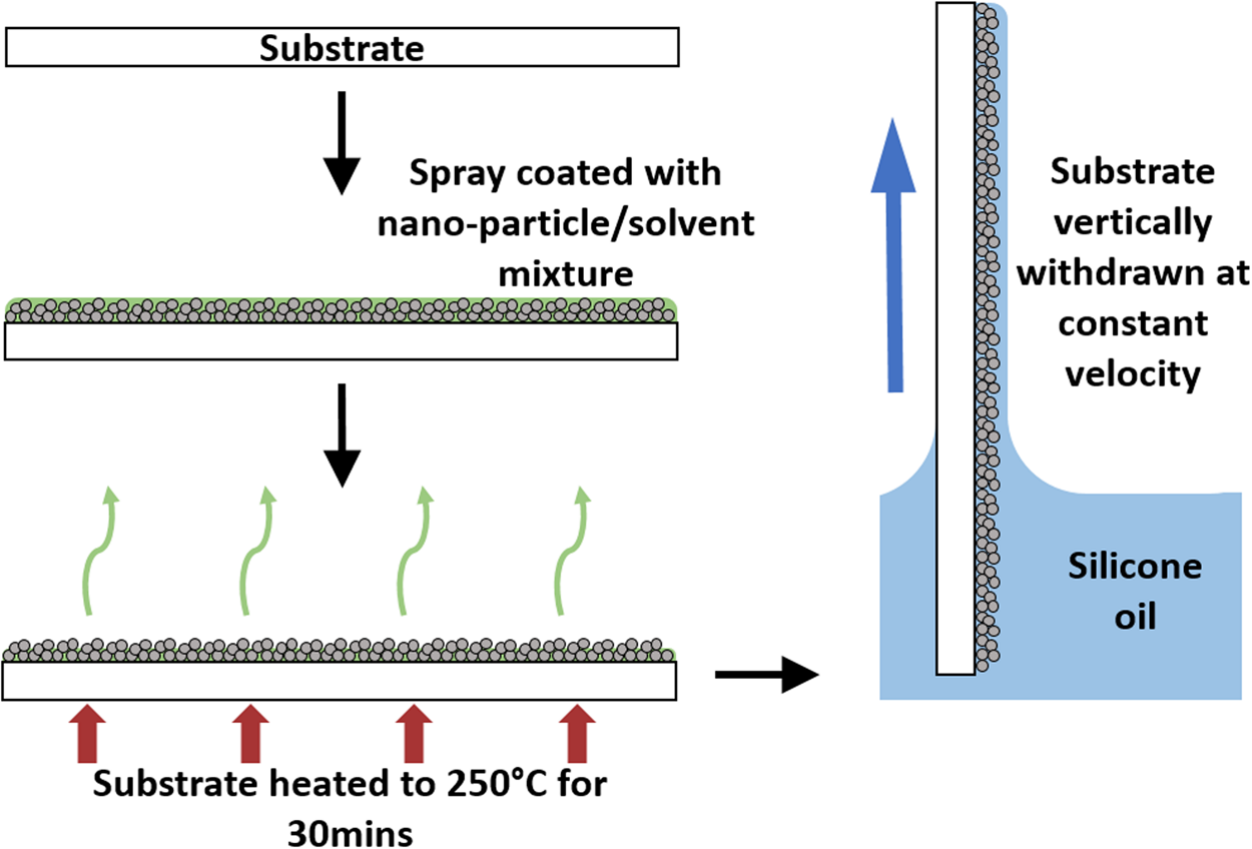
Schematic of the SLIPS fabrication process.

The sample preparation process is summarized in Fig. 5. To characterize the spray coating process, we first fabricated flat samples. Glass slides, 75 mm by 25 mm were placed in a 2%vol solution of Decon 90 surface decontaminant and deionized water and sonicated in an ultrasonic bath for 15 minutes. They were then rinsed in deionized water and finally in isopropanol (IPA). The clean glass substrates were then inclined at an angle of 45° and a nanoparticle/solvent mixture known as Glaco^®^ Mirror Coat, a commercially available aerosol for creating superhydrophobic coatings, was sprayed onto the substrates from top to bottom in one pass. The Glaco^®^ mirror coat contains functionalized silica nanoparticles suspended in a propanol/liquefied petroleum gas mixture. The application of the superhydrophobic nanoparticle creates a porous particle network which is oleophilic. These properties allow the lubricating oil to wick into the porous structure because of its preference to wet the particles. Oleophilic surfaces are often hydrophobic and this was confirmed by measuring the contact angles for both silicone oil and water.

Depending on the substrate material, there are two choices for the drying stage, a high or a low temperature option. For the low temperature option, sprayed substrates were left untouched for 3 hours for the solvent within the mixture to completely evaporate. For the high temperature option, the substrates were left for 5 minutes and then baked at 250 °C for 30 mins. This shortens the duration of the evaporation process by driving off the solvent, using elevated temperatures. This option requires the substrates to be able to withstand such high temperatures. Once the drying stage has completed, and the high temperature surfaces were allowed to return to room temperature, it is possible to repeat the process and apply another layer of the nanoparticle coating. Both methods result in the same surface coating with no noticeable effects to the wetting properties of the substrates. In this report, the low temperature method was used to coat the glass slides and the glass tube, whereas the high temperature method was used to coat the #120,#150,#250,#400 and #500 stainless steel mesh, with all surfaces receiving 5 applications of the spray coating.

After spray coating, all of the coated substrates were dip-coated with silicone oil (Sigma-Aldrich). The silicone oil has a viscosity of 20 cSt and a surface tension of 20.6 mNm^−1^. The samples were immersed in a bath of silicone oil and withdrawn vertically at a constant speed of 0.1 mm/s. The constant withdrawal speed was achieved using a Fisnar F4200N, a compact benchtop robot that is capable of linear travel, accurate to ±0.02 mm/s. The controlled withdrawal speed ensures a consistent thickness of lubricant on the surface^39^ and we have shown in previous work^40^ that this method also allows for control of the oil thickness above the imbibed structure.

Drop shape analysis was performed on the spray coated substrates, using a Krüss DSA30, a drop shape analyzer, in order to measure the advancing and receding contact angles of water droplets on the developing surface coating (Fig. 1(a)). The measurements were performed on 5 different substrates, which had been coated between 1 and 5 times with the spray method, in order to determine average advancing and receding contact angles for the nanoparticle surface.

To characterize the surface, scanning electron microscopy was performed, using a FEG Tescan MIRA3 (Fig. 1(b–k)) after coating the samples with a 5 nm conductive layer of platinum. We used cross-section images to determine the average thickness with increasing number of coatings.

To determine the effectiveness of the SLIP coating we measured the sliding properties of water droplets on the SLIP surfaces using a Krüss DSA30, equipped with a tilt table capable of 0.1° increments. The Krüss DSA30 was initially carefully levelled using a Level Developments certified Engineer’s workshop level, sensitive to 0.05 mm/m (equivalent to a tilt of 0.003°). Measurements were performed on 5 different substrates, which had been coated between 1 and 5 times, to determine an average sliding angle for the lubricant impregnated surfaces.

To determine the robustness of the lubricating silicone oil layer when applied to the mesh, 250 stainless steels mesh SLIPS was fabricated and subjected to flow conditions generated by a constant flow rate setup^32^. The mesh SLIPS samples were positioned along the center of a 8 mm diameter pipe, through which water flowed. Initially, two viscosities of silicone oil were used to fabricate SLIPS materials, 20 cSt and 1000 cSt (Sigma-Aldrich), that were tested in a turbulent flow, Re = 8200 ± 500, to determine the longevity of the SLIPS coating to maintain a sliding angle of >5°. Subsequently, #250 SLIPS mesh, coated with 1000 cSt silicone oil was tested at both a laminar flow rate, Re = 1550 ± 50, and a turbulent flow rate, Re = 4000 ± 300, with the sliding angle of 2 µl water droplets being measured at 15 minute intervals.

## Supplementary information

Supplementary Information

Supplementary Video 1

Supplementary Video 2

## Data Availability

All data generated or analysed during this study are included in this published article (and its Supplementary Information Files).
