# Supplementary material for: Double-sided slippery liquid-infused porous materials using conformable mesh
Source: Sci Rep. 2019 Sep 16;9:13280. doi: 10.1038/s41598-019-49887-3 (PMC6746700; doi:10.1038/s41598-019-49887-3)
Supplement: Supplementary file 1 — Supplementary Information [file 41598_2019_49887_MOESM1_ESM.docx]

Double-sided slippery liquid-infused porous materials using conformable mesh

Nicasio R. Geraldi^1a^, Jian H. Guan^2^, Linzi E. Dodd^2^, Pietro Maiello^2^, Ben B. Xu^2^, David Wood^2^, Michael I. Newton^1^, Gary G. Wells^2^, Glen McHale^2^

^1^School of Science and Technology, Nottingham Trent University, Nottingham NG11 8NS, U.K.

*^2^*Smart Materials and Surfaces Laboratory, Faculty of Engineering and Environment, Northumbria University, Newcastle upon Tyne, NE1 8ST, U.K.

^a^ Corresponding Author:

Email: nicasio.geraldi02@ntu.ac.uk

Supplementary Information

Videos, Supplementary Video 1 and Supplementary Video 2, show droplets of distilled water on stainless steel mesh that has been transformed into slippery materials. Supplementary Video 1 shows droplets sliding on both sides of a #500 mesh SLIPS and Supplementary Video 2 shows droplets travelling around a loop made from #150 mesh SLIPS.
